# Data blinding for the nEDM experiment at PSI

**DOI:** 10.1140/epja/s10050-021-00456-1

**Published:** 2021-04-28

**Authors:** N. J. Ayres, G. Ban, G. Bison, K. Bodek, V. Bondar, E. Chanel, P.-J. Chiu, C. B. Crawford, M. Daum, S. Emmenegger, L. Ferraris-Bouchez, P. Flaux, Z. Grujić, P. G. Harris, N. Hild, J. Hommet, M. Kasprzak, Y. Kermaïdic, K. Kirch, S. Komposch, A. Kozela, J. Krempel, B. Lauss, T. Lefort, Y. Lemiere, A. Leredde, P. Mohanmurthy, A. Mtchedlishvili, O. Naviliat-Cuncic, D. Pais, F. M. Piegsa, G. Pignol, M. Rawlik, D. Rebreyend, I. Rienäcker, D. Ries, S. Roccia, D. Rozpedzik, P. Schmidt-Wellenburg, A. Schnabel, R. Virot, A. Weis, E. Wursten, J. Zejma, G. Zsigmond

**Affiliations:** 1 Institute for Particle Physics and Astrophysics, ETH Zürich, Zürich, Switzerland; 2 Department of Physics and Astronomy, University of Sussex, Falmer, Brighton UK; 3 Normandie Université, ENSICAEN, UNICAEN, CNRS/IN2P3, LPC Caen, Caen, France; 4 Paul Scherrer Institute, Villigen, Switzerland; 5 M. Smoluchowski Institute of Physics, Jagiellonian University in Krakow, Kraków, Poland; 6 Instituut voor Kern- en Stralingsfysica, Katholieke Universiteit Leuven, Leuven, Belgium; 7 University of Bern, Albert Einstein Center for Fundamental Physics, Laboratory for High Energy Physics, Bern, Switzerland; 8 Department of Physics and Astronomy, University of Kentucky, Lexington, USA; 9 Univ. Grenoble Alpes, CNRS, Grenoble INP, LPSC-IN2P3, Grenoble, France; 10 Physics Department, University of Fribourg, Fribourg, Switzerland; 11 H. Niewodniczanski Institute of Nuclear Physics, Polish Academy of Sciences, Kraków, Poland; 12 Department of Chemistry - TRIGA site, Johannes Gutenberg University Mainz, Mainz, Germany; 13 Institut Laue-Langevin, Grenoble, France; 14 Physikalisch Technische Bundesanstalt, Berlin, Germany; 15 Present Address: Institute of Physics Belgrade, Belgrade, Serbia; 16 Present Address: CERN, Geneva, Switzerland

## Abstract

Psychological bias towards, or away from, prior measurements or theory predictions is an intrinsic threat to any data analysis. While various methods can be used to try to avoid such a bias, *e.g. *actively avoiding looking at the result, only data blinding is a traceable and trustworthy method that can circumvent the bias and convince a public audience that there is not even an accidental psychological bias. Data blinding is nowadays a standard practice in particle physics, but it is particularly difficult for experiments searching for the neutron electric dipole moment (nEDM), as several cross measurements, in particular of the magnetic field, create a self-consistent network into which it is hard to inject a false signal. We present an algorithm that modifies the data without influencing the experiment. Results of an automated analysis of the data are used to change the recorded spin state of a few neutrons within each measurement cycle. The flexible algorithm may be applied twice (or more) to the data, thus providing the option of sequentially applying various blinding offsets for separate analysis steps with independent teams. The subtle manner in which the data are modified allows one subsequently to adjust the algorithm and to produce a re-blinded data set without revealing the initial blinding offset. The method was designed for the 2015/2016 measurement campaign of the nEDM experiment at the Paul Scherrer Institute. However, it can be re-used with minor modification for the follow-up experiment n2EDM, and may be suitable for comparable projects elsewhere.

## Introduction

The electric dipole moment (EDM) of the neutron is a fundamental observable in particle physics that may directly relate to the observed dominance of matter over antimatter in the Universe. It has been sought experimentally for almost seven decades, with ever-improving sensitivity, but to date all results have been compatible with zero [1–5]. Many theories beyond the Standard Model naturally predict non-zero values that are close to current experimental sensitivities [6–8]. Thus, depending upon their outlook, scientists analysing the data from EDM experiments may be biased unintentionally towards a result that favours their own expectations of either seeing, or not seeing, a statistically significant signal. Data blinding removes this psychological bias and, if applied properly, does not introduce any other bias. In experimental particle physics blinding has been used quite commonly for many years [9], but it has not previously been applied to any neutron EDM measurement.

In general, at least two different types of blinding can be distinguished:

1. Data corresponding to a region of interest is withheld from the analysis team, or, correspondingly, “fake” events can be added to obscure the signal. This is often the case in discovery experiments. See, *e.g. *(not the latest but representative) searches for rare decays [10], dark matter [11] or gravitational waves [12].
2. For precision experiments the observable of interest can be scaled by an unknown factor [13], or in some cases, an unknown offset can be added to the observable [14].

The latter is applicable to EDM experiments, and it is the approach that we have adopted for the nEDM experiment at the Paul Scherrer Institute (PSI) [15]. In deciding to modify the observable, one can choose to do so either by changing an aspect of the experiment itself, or by modifying the data *post hoc*. The latter has the advantage that it does not change or corrupt the experiment, and a hidden set of original data can be stored securely. Thus, if the blinding were to affect the data quality in any unforeseen way, *e.g. *by reducing the sensitivity or by introducing a new bias, the original data can still be used in the knowledge that the final result is unaffected by any systematic effects that may have been introduced through blinding. It should be mentioned that the data blinding described in this publication targets solely the psychological bias during data analysis. Any other potential bias of the measurement must be addressed by other means.

## Experimental overview

**Fig. 1:**
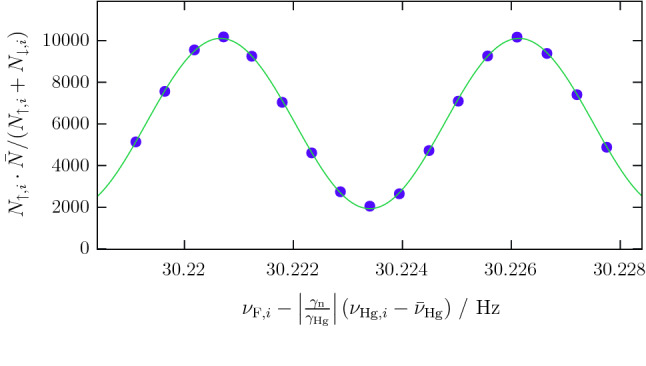
Measured neutron counts plotted versus spin-flip frequency. Both quantities are corrected for fluctuations, as indicated in the axis labels. A sinusoidal curve with offset is fitted to the data points. $\bar{\nu }_\mathrm {Hg}$ is the average reading of the mercury co-magnetometer. Both averages used in this plot are calculated from the measurements shown in this graph

In nEDM experiments the observable of interest is the dependence of the neutrons’ Larmor precession frequency upon an applied static electric field [15]. In most experiments to date the frequency measurement has been based on Ramsey’s technique of separated oscillatory fields [16]. In the experiment at PSI, polarized ultracold neutrons (UCN) were stored in a cylindrical vessel within a stable and highly uniform magnetic field parallel to the axis of the storage vessel. The two Ramsey spin-flip pulses, in phase with one another and each capable of inducing a $\pi /2$ spin rotation, were applied via a transverse rotating magnetic field. Between the two pulses, the neutron spins precessed freely. If the spin-flip frequency was perfectly in resonance with the Larmor frequency of the neutrons, the neutrons would undergo a $\pi$ spin-flip by the end of the procedure. If not, the accumulated phase difference – a highly sensitive measure of the difference between the Larmor and reference frequencies – would result in a partial spin-flip. Following this procedure, the neutrons were counted in a spin-sensitive detector. By repeating such measurements while scanning the reference frequency, and plotting the final neutron spin state versus that frequency, a Ramsey fringe pattern emerges as shown in Fig. 1. For a non-zero EDM value the pattern will shift horizontally when the electric field direction is reversed, which is done periodically.

An adiabatic fast-passage spin-flipper, referred to as SF1, was present at the entrance to the apparatus. When activated, it inverted the initial neutron spin orientation. This was used to investigate the influence of systematic effects. Regular changes of the magnetic field orientation and a variation of the magnetic field gradient serve the same purpose.

The 2015/16 data-taking campaign at PSI [15] employed a UCN storage volume of 22 litres, and the (highly uniform) magnetic field had a magnitude of approximately 1$\upmu$T. At the heart of each single measurement, known as a “cycle”, were the two $\pi /2$ spin-flip pulses; these had a frequency of $\nu _{\mathrm {F},i} \approx {30}{\hbox {Hz}}$ and were applied for a duration of $t={2}{\hbox {s}}$ each. They were separated by a free precession period of $T={180}{\hbox {s}}$. Subsequent to this the neutrons were released from the storage volume and were counted in a spin-sensitive detector, yielding up to 20000 UCN per cycle.

The detector contained two separate branches, each consisting of a controllable adiabatic fast-passage spin-flipper, a magnetized spin-analysing foil, and a set of nine ^6^Li based neutron detectors that were read out via photomultiplier tubes (PMTs) [17, 18]. This dual readout enabled the simultaneous measurement both of spin-up and of spin-down neutrons. Every time the current in one of the PMTs exceeded a certain threshold, the recording of an “event” was triggered. Typically these events were caused by single neutrons, but some were due to incident photons. The timestamp, integrated charge and detector channel of every event were recorded in the data files. A set of consecutive cycles carried out with a stable magnetic field configuration, but with variation of the applied electric field, was called a “run”. During a run, lasting for up to several days and typically consisting of several hundred cycles, the configuration of the spin-flippers in the detector branches was reversed every four cycles, and the entrance spin-flipper status (the spin orientation of the neutrons entering the storage volume) was changed every 112 cycles. Within a run, eight cycles with no electric field were followed by 48 cycles with high voltage of a given polarity. Thus, both electric field polarities were applied between each change of the entrance spin-flipper state.

### Formal description

The $\pi /2$ Ramsey spin-flip pulses of frequency $\nu _{\mathrm {F},i}$ that are applied in a particular cycle *i* cause a change in the relative proportions of spin-up and spin-down neutrons detected, with the position on the curve of Fig. 1 being determined by the phase

1 $$\begin{aligned} \phi _i = \frac{(\nu _{\mathrm {F},i}-\nu _{\mathrm {L}})}{\varDelta \nu }\pi . \end{aligned}$$

Here $\nu _{\mathrm {L}}$ is the Larmor frequency, and the fringe width $\varDelta \nu$ is

2 $$\begin{aligned} \varDelta \nu = \frac{1}{2\left( T+4\,t/\pi \right) }, \end{aligned}$$

where *T* is the free-precession time and *t* the duration of each spin-flip pulse.

The true numbers of neutrons of each spin state, $N^\prime _{\uparrow ,i}$ and $N^\prime _{\downarrow ,i}$, within the storage volume just before they are guided to their respective detectors are

3 $$\begin{aligned} N^\prime _{\uparrow ,i}= & {} \frac{N^\prime _i}{2} \left( 1 - \alpha ^\prime \cos \phi _i \right) \end{aligned}$$

4 $$\begin{aligned} N^\prime _{\downarrow ,i}= & {} \frac{N^\prime _i}{2} \left( 1 + \alpha ^\prime \cos \phi _i \right) \ , \end{aligned}$$

where $N^\prime _i$ is the total number of neutrons in the chamber after the precession and $\alpha ^\prime$ describes the true visibility after the precession; note that $\alpha ^\prime$ has a negative sign when SF1 is enabled.

The neutrons then fall through a polarisation analyser with spin selectivities $p_\mathrm {A}$ and $p_\mathrm {B}$ to reach the detectors that operate with efficiencies $\epsilon _\mathrm {A}$ and $\epsilon _\mathrm {B}$. Thus, the numbers of neutrons measured are

5 $$\begin{aligned} N_{\uparrow ,i}= & {} \big ( N^\prime _{\uparrow ,i} \, p_\mathrm {A} + N^\prime _{\downarrow ,i} (1-p_\mathrm {A}) \big ) \epsilon _\mathrm {A} \end{aligned}$$

6 $$\begin{aligned} N_{\downarrow ,i}= & {} \big ( N^\prime _{\downarrow ,i} \, p_\mathrm {B} + N^\prime _{\uparrow ,i} (1-p_\mathrm {B}) \big ) \epsilon _\mathrm {B} \ . \end{aligned}$$

In this model the efficiency of the spin-flippers is neglected since it is very close to unity.

“Spin up” ($\uparrow$) refers to neutrons with the spin polarisation antiparallel to the magnetic field $B_0$, and therefore with the magnetic moment parallel to the field. They are also known as “high field seekers”. When SF1 is off, this is the state in which they enter the bottle and is thus their state before the Ramsey sequence is applied.

**Fig. 2:**
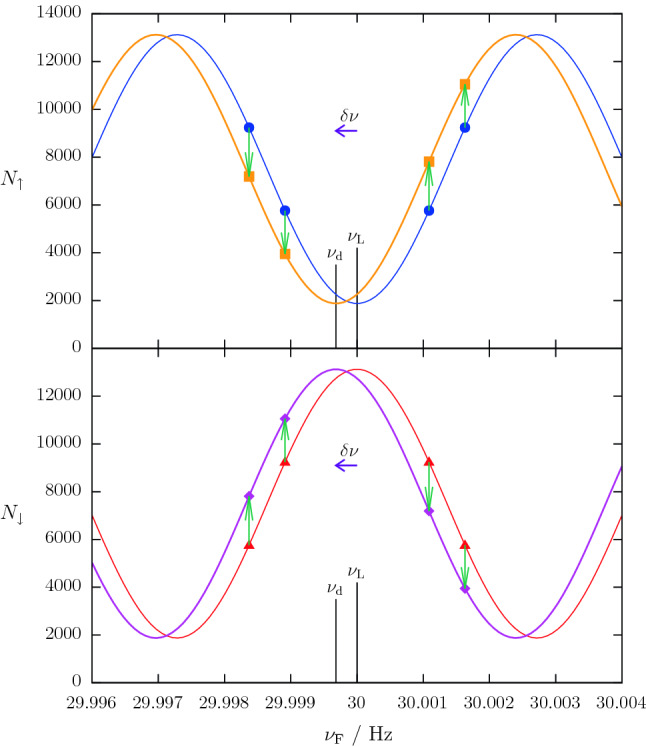
Simulated neutron counts plotted versus applied spin-flip frequency $\nu _\mathrm {F}$. The transfer of a small number of neutrons (green arrows) from their initially recorded state, *e.g. *counts $N_\uparrow$ (blue circles), corresponding to the original Larmor frequency $\nu _{\mathrm {L}}$, to the other spin state creates the blinded data points (orange squares). If this is done systematically and proportionally to the electric field, one can extract from the resulting dashed orange line a different Larmor frequency $\nu _\mathrm {d}=\nu _{\mathrm {L}}+\delta \nu$. The frequency shift by $\delta \nu$ (violet arrow) represents a false EDM signal *d* given by Eq. (7). For the detector arm counting the opposite spin state, *e.g.* $N_\downarrow$, the corresponding shift leads from the solid red to the dotted magenta curve. This yields the same false EDM signal. In the case where SF1 is active, all points and lines must be mirrored in a horizontal line at $N=7500$. For clarity the strongly exaggerated values $\left| \sin \phi \right| =0.951$ and $d={3\times 10^{-23}}\,{\textit{e}\,cm}$ have been used here

A fit of Eqs. (1)–(6) to the data of all cycles of a run yields $\nu _L$ and $\alpha ^\prime$, while *T*, *t* and all $\nu _{\mathrm {F},i}$ are known parameters. The steepest part of the slope, *i.e. *where $\phi _i\approx \pm 90^{\circ }$, is most sensitive to variations of the Larmor frequency. Thus the spin-flip frequencies were configured to operate sequentially at four distinct frequencies, the so-called working points (corresponding to the horizontal positions of the arrows in Fig. 2). These were detuned from the steepest point by about 5% of the fringe width in order to have some sensitivity also to further experimental parameters such as the visibility (effectively, the amplitude of the sinusoidal curve) and, in a more detailed analysis described in Sect. 3.2.2, the asymmetry of the detector efficiency.

In the presence of an nEDM *d* and an applied electric field $\mathbf {E}$ collinear to the magnetic field $\mathbf {B}$, the resonant frequency $\nu _{\mathrm {L}}$ shifts by

7 $$\begin{aligned} \delta \nu = 2\,d\, \mathbf {E} \cdot \frac{\mathbf {B}}{B} /h, \end{aligned}$$

where *h* is Planck’s constant. Note that the $\mathbf {B}/B$ term is required only to obtain the appropriate sign.

Any change of the amplitude of the ambient magnetic field causes a corresponding change of the Larmor frequency. A mercury co-magnetometer was used to compensate for magnetic-field fluctuations by using the ratio of the measured frequencies $\mathcal {R}=\nu _\mathrm {n}/\nu _\mathrm {Hg}$ [19]. Correspondingly, Eq. (1) is altered as shown in Eq. (12). However, although the (thermal) mercury atoms populate the storage cell rather uniformly, the UCN have such low kinetic energies that under the influence of Earth’s gravitational field their mean vertical position lies a few mm below that of the mercury. Any vertical gradient of the magnetic field therefore results in a different average value of the magnetic field for the two species. This in turn leads to a small shift in the mercury-corrected neutron Larmor frequency. For a given vertical gradient, this shift is in opposite directions for the two different orientations (up vs. down) of the main magnetic field. Furthermore, there is a systematic effect leading to a significant false EDM arising from a conjunction of the vertical magnetic-field gradient and the relativistic motional magnetic field seen by the mercury atoms (in particular) as they move through the electric field [20]. It was therefore necessary to interpolate the measured nEDM results to zero vertical magnetic-field gradient. Since there was no absolute gradiometer, small magnetic-field gradients were applied using trim coils in order to determine the situation at zero gradient from the intersection of the two curves arising from the two magnetic-field directions [15, 20, 21]. It is important to state that a blinding nEDM offset does not interfere with the interpolation of the curves.

## Data blinding

### Blinding concept

Any offset-based blinding method for an nEDM experiment must shift the measured Larmor frequency proportionally to the electric field, ideally while leaving all other observables unaltered. The following blinding procedures – each of which would have served to mimic an EDM within the usual analysis strategies – were briefly considered by our collaboration:

- Apply a modified spin-flip frequency with respect to the recorded value during the experiment. However, this would modify the experiment in an insidious manner as the change in actual physical conditions applied would be correlated to the electric field changes. This could therefore potentially introduce systematic effects, and, additionally, it would be irreversible. One would therefore have no possibility to investigate (or remove) it *a posteriori*.
- Register a shifted spin-flip frequency with respect to the one actually applied. This was not practicable in our case because of the finite resolution with which the frequency could be set. Furthermore, since this frequency was calculated from the mercury co-magnetometer reading of the previous cycle, this method would have required subtle alterations to all magnetometer readings in order to avoid the possibility of the shift being revealed through simple comparison. In our case this would have meant consistently adjusting a total of 16 magnetometer readings (one mercury and 15 caesium) [15, 22] – a daunting task.

Although it does not apply to the current measurement it is also worth noting that these techniques could not be used in double-cell nEDM experiments, since common Ramsey spin-flip pulses would be applied to the entire assembly but the required shifts in each of the two cells would be in opposite directions.

There are various other observables that could have been modified but which would not have given exactly the same appearance as an EDM signal, and it would thus have been fairly trivial to identify them as fake signals. Note that manipulating the value of the electric field cannot be used to introduce a blinding offset.

The remaining variable that can usefully be modified is the number of neutrons counted in each spin state. The primary difficulty in this case is that, since the size of the required shift itself depends upon the number of neutrons counted, a partial but automatic preliminary analysis of the data must first be carried out. Furthermore, in order to deliver blinded data to the analysis teams as early as possible this preliminary analysis must be undertaken in real time in a manner that is fully defined before starting the actual data-taking campaign. Ultimately all of this proved to be manageable, and the approach was therefore adopted for the nEDM experiment. Its implementation will be described in detail in the following sections.

### Algorithm

The blinding algorithm operates in a stepwise manner. First the necessary parameters are extracted from a full run (Sect. 3.2.2). Then the position of each cycle on the Ramsey curve, the so-called working point, is determined (Sect. 3.2.3), before the number of neutrons in each cycle can be modified (Sect. 3.2.4).

#### Calculation of the number of neutrons to be transferred

In order to generate an $\mathbf {E}$-field dependent frequency shift a small number of spin-up neutrons have to be reclassified as spin down, or *vice versa*, as illustrated in Fig. 2.

We follow Eqs. (1), (5), (6) and (7) as well as the first-order Taylor expansion $\delta N= \left( \frac{\mathrm {d}}{\mathrm {d}\phi } N \right) \left( \frac{\mathrm {d}}{\mathrm {d}\nu _\mathrm {L}} \phi \right) \delta \nu$ to find the number of neutrons that need to change state:

8 $$\begin{aligned} \delta N_{\uparrow ;i}= & {} \epsilon _\mathrm {A} \frac{N_i^\prime }{2} \alpha ^\prime \left( 2p_\mathrm {A} -1 \right) \left( \sin \phi _i \right) \left( \frac{\mathrm {d}}{\mathrm {d}\nu _\mathrm {L}} \phi \right) \delta \nu \end{aligned}$$

9 $$\begin{aligned}= & {} - \epsilon _\mathrm {A} \frac{N_i^\prime }{2} \alpha ^\prime \left( 2p_\mathrm {A} -1 \right) \left( \sin \phi _i \right) \frac{\pi }{\varDelta \nu }\frac{2 \, d \, \mathbf {E} \cdot \mathbf {B} / B }{h}; \end{aligned}$$

10 $$\begin{aligned} \delta N_{\downarrow ;i}= & {} + \epsilon _\mathrm {B} \frac{N_i^\prime }{2} \alpha ^\prime \left( 2p_\mathrm {B} -1 \right) \left( \sin \phi _i \right) \frac{\pi }{\varDelta \nu }\frac{2 \, d \, \mathbf {E} \cdot \mathbf {B} / B }{h}.\nonumber \\ \end{aligned}$$

Note that $\mathbf {E}$ and $\mathbf {B}$ have to be parallel or antiparallel, and that the sign of $\alpha ^{\prime }$ can be negative depending upon the state of SF1.

It is convenient to introduce the total number of measured counts per cycle $N_i= \frac{ \epsilon _\mathrm {A} +\epsilon _\mathrm {B} }{2} N_i^\prime$ and its average over a run $\bar{N}=\,<\!N_i\!>$. In light of the performance of the detectors [17, 23]) (see also Sect. 3.8.2), it is useful to note that, to a good approximation, $\epsilon _\mathrm {A}=\epsilon _\mathrm {B}$. Furthermore, since the data show that $\left( 2p_\mathrm {A} -1 \right) /\left( 2p_\mathrm {B} -1 \right) -1 \approx 0.15\%$ the performance of the spin analysers can be assumed to be equal for the two spin states, *i.e.* $p_\mathrm {A}=p_\mathrm {B}$. Thus the measured visibility becomes $\alpha =\alpha ^\prime (2 p_\mathrm {A} -1)$, again with the usual caveat that it is negative when SF1 is enabled. Therefore,

11 $$\begin{aligned} \delta N_{\uparrow ,\downarrow ;i} = \mp N_i \frac{\pi \alpha }{\varDelta \nu }\frac{d \, \mathbf {E} \cdot \mathbf {B} / B}{h} \sin \phi _i. \end{aligned}$$

The implications of removing the assumptions $\epsilon _\mathrm {A}=\epsilon _\mathrm {B}$ and $p_\mathrm {A}=p_\mathrm {B}$ will be discussed below.

Typical values for the nEDM experiment are $N_i$ = 15,000, $\left| \sin \phi \right|$ = 0.99, $\alpha$ = 0.75, *T* = 180s, *t* = 2s, and *E* = 11kV/cm. Thus an EDM offset of ${1.0\times 10^{-25}}$ *e* cm would require a shift of about 3.39 neutrons in each cycle. Bearing in mind that the neutron has a negative magnetic moment, if $\mathbf {B}$ and $\mathbf {E}$ are parallel a positive nEDM would *reduce* the precession frequency. This would shift the Ramsey curves towards smaller frequencies, meaning that neutrons measured at a working point above the resonant frequency would shift from the spin-down detector arm to the spin-up. Neutrons that are measured at a working point below the resonant frequency would correspondingly shift from the spin-down to the spin-up detector arm. Figure 2 illustrates this reclassification and the resulting shift.

Obviously, it is impossible to shift a non-integer number of neutrons in a single cycle. One could simply round the number, but this would effectively cause a granularity of $\sim$ ${3\times 10^{-26}}$ *e* cm in the available blinding offsets. However, one can add to $\delta N$ a small random number with a normal distribution, and round the sum to the nearest integer number. The choice of the width of this normal distribution had to strike a balance between two competing factors: On the one hand, a small width does not sufficiently smooth the granularity. On the other hand, a large width adds noise to the neutron counts and thus to the blinded nEDM value. A suitable compromise was found that used a standard deviation of 2 counts. In this case the granularity is sufficiently suppressed so that the result differs from a flat distribution by less than $10^{-7}$. An improved method will be suggested in Sect. 6.

As mentioned above, this algorithm assumes the same $\bar{N}$ and $\alpha$ for each of the two spin states. If this were not to be the case, a direct transfer of neutrons from one spin state to the other would not be appropriate. Instead, one would have to analyse and treat the two states separately, and neutrons would have to be added to or deleted from the spin-up and spin-down arrays as required. While this would be trivial if the neutron data were to consist merely of a simple sum of counts per cycle, it is a substantial effort for a more detailed data format such as ours, which lists charge and time per event.

#### Determination of $\alpha$ and detector asymmetry

As is evident from Eq. (11), before the data can be blinded one has to determine $\alpha$ and $\nu _\mathrm {L}$. While $\alpha$ is sufficiently constant throughout an entire run, $\nu _\mathrm {L}$ might change from cycle to cycle and must be corrected with the field values recorded by the mercury co-magnetometer. We therefore refer to it as $\nu _{\mathrm {L},i}$ and write

12 $$\begin{aligned} \nu _{\mathrm {L},i} = \left| \frac{\gamma _\mathrm {n}}{\gamma _\mathrm {Hg}}\right| \nu _{\mathrm {Hg},i} - \frac{\varPhi }{\pi } \varDelta \nu , \end{aligned}$$

where $\gamma _\mathrm {n}$ and $\gamma _\mathrm {Hg}$ are the gyromagnetic ratios of the neutron and mercury respectively, $\nu _{\mathrm {Hg},i}$ is the frequency obtained from the mercury co-magnetometer, and the phase $\varPhi$ accommodates any difference in the average magnetic field sampled by the two species. The ratio of gyromagnetic ratios was measured in a previous experiment [24]. For the blinding algorithm, a fixed value of

$$\begin{aligned} \left| \frac{\gamma _\mathrm {n}}{\gamma _\mathrm {Hg}}\right| = 3.8424574 \end{aligned}$$

was used. As magnetic field gradients were not changed during a run, $\varPhi$ kept the same value throughout all cycles of the run.

Equations (5) and (6) can be rewritten as

13 $$\begin{aligned}&\frac{N_{\uparrow ,i} - N_{\downarrow ,i}}{N_{\uparrow ,i} + N_{\downarrow ,i}} \nonumber \\&= \frac{\epsilon _\mathrm {A}-\epsilon _\mathrm {B}-\alpha ^\prime \left( \epsilon _\mathrm {A} (2 p_\mathrm {A} -1) + \epsilon _\mathrm {B} ( 2p_\mathrm {B} -1) \right) \cos \phi _i}{ \epsilon _\mathrm {A}+\epsilon _\mathrm {B}+\alpha ^\prime \left( -\epsilon _\mathrm {A} (2 p_\mathrm {A} -1) + \epsilon _\mathrm {B} ( 2p_\mathrm {B} -1) \right) \cos \phi _i} \end{aligned}$$

14 $$\begin{aligned}&\approx \frac{\epsilon _\mathrm {A}-\epsilon _\mathrm {B}}{\epsilon _\mathrm {A}+\epsilon _\mathrm {B}} - \alpha \cos \phi _i, \end{aligned}$$

where use is made of the approximations, justified by data, $p_\mathrm {A} \approx p_\mathrm {B}$ and $\epsilon _\mathrm {A}\approx \epsilon _\mathrm {B}$. The latter also implies that $\epsilon _\mathrm {A}-\epsilon _\mathrm {B} \ll \epsilon _\mathrm {A} + \epsilon _\mathrm {B}$.

We define $A_m=\frac{\epsilon _\mathrm {A}-\epsilon _\mathrm {B}}{\epsilon _\mathrm {A}+\epsilon _\mathrm {B}}$, which is the detector asymmetry originating from the slightly different efficiencies of the two detector arms counting the two spin states. Equations (12) and (1) are used to rewrite Eq. (14) as

15 $$\begin{aligned} \frac{N_{\uparrow ,i} - N_{\downarrow ,i}}{N_{\uparrow ,i} + N_{\downarrow ,i}} = f\left( \nu _{\mathrm {F},i} - \left| \frac{\gamma _\mathrm {n}}{\gamma _\mathrm {Hg}}\right| \nu _{\mathrm {Hg},i} \right) , \end{aligned}$$

where we have defined the function

16 $$\begin{aligned} f(x) = A_m - \alpha \cos \left( \frac{\pi }{\varDelta \nu } x + \varPhi \right) . \end{aligned}$$

The independent variable *x* is beneficial for the fit algorithm, since it can be calculated from the observables for each cycle. It represents the difference between the applied spin-flip frequency and the neutron resonance frequency. The parameter estimation of $A_m$, $\alpha$ and $\varPhi$ is carried out by fitting the data of a full run to Eq. 15. Every four cycles the spin-flipper states of both detector arms were inverted by activating and deactivating spin-flipper coils that are mounted inside the detector arms [17, 18, 23]. This results in a “normal” and an “inverted” configuration, with asymmetries $A_{\mathrm {N}}$ and $A_{\mathrm {I}}$ respectively. Both values are almost constant throughout a run. They were retained as fit parameters in order to accommodate long-term changes. Consequently the data contain two collated subsets, and the fit had to be conducted as a simultaneous fit within which $\alpha$ and $\varPhi$ were common parameters while $A_{\mathrm {N}}$ and $A_{\mathrm {I}}$ applied only to the respective partial data sets.

#### Determination of the Ramsey phase $\phi _i$

After having carried out the fit on the full run, Eq. (11) was used to calculate the number of neutrons to be transferred for each cycle. However, it was still necessary to determine $\phi _i$. This could be done either via Eq. (14),

17 $$\begin{aligned} \cos \phi _i = \frac{1}{\alpha } \left( \frac{N_{\uparrow ,i} - N_{\downarrow ,i}}{N_{\uparrow ,i} + N_{\downarrow ,i}} - A_m \right) , \end{aligned}$$

or via Eq. (1),

18 $$\begin{aligned} \phi _i = \frac{ \nu _{\mathrm {F},i}- \left| \frac{\gamma _\mathrm {n}}{\gamma _\mathrm {Hg}}\right| \nu _{\mathrm {Hg},i} }{\varDelta \nu }\pi +\varPhi . \end{aligned}$$

The first variant was implemented here, as it is more robust in instances where in a single cycle the co-magnetometer provides a reading with a large uncertainty which would potentially lead to a wrong blinding of that cycle. Note that this variant also uses Eq. (18) to determine the sign of $\phi _i$.

#### Transferring neutrons

The data files are an event-driven list where each entry consists of a time stamp, the integrated charge recorded at the time, and the identification number of the photomultiplier tube that observed the event [18]. If the integrated charge exceeds a certain threshold then the event is classified as a neutron detection. Each of the two detector arms, one per spin state, contains a set of nine PMTs, which are sequentially numbered from 0 to 17 with 0–8 in the first arm and 9–17 in the second. In order to reclassify the spin of a neutron it is therefore sufficient to take the PMT number of that event, add 9 and carry out a modulo 18 operation. A neutron that is to be transferred is chosen by randomly selecting an event from the list counted in the correct detector arm, and then checking whether it is suitable to be moved simply and cleanly across: the requirement is that there must be a minimum separation in time between the event in question and the previous and subsequent events. We apply this condition to both the source and the recipient channel. The reason is to avoid the transfer of events for which the charge is split between neighbouring PMTs, or where the baseline correction algorithm has or would have to modify the charge [25]. If the event is not suitable, another randomly chosen event is tested until an appropriate one is found. The charge threshold for the neutron identification was investigated before the measurement campaign. Since the analysis teams could in principle have found and used a slightly different value, the blinding used a 60% higher threshold. We had carefully estimated that the change was small enough for the total number of events occurring between the two charge values to be sufficiently low to yield a statistical uncertainty that would be too large to make a useful prediction of the blinding offset.

### Choice of the blinding offset

Obviously, the value of the blinding offset must be kept secret from the analysis teams. In order to avoid providing any indirect psychological bias as to its value, it is necessary to choose it randomly from a distribution that allows a wide range of such values. It is convenient for its modulus to be larger than the known upper limit of the nEDM, since this allows a “sanity check” of having a sign that can be confirmed for consistency prior to publication of results (see Sect. 5).

**Fig. 3:**
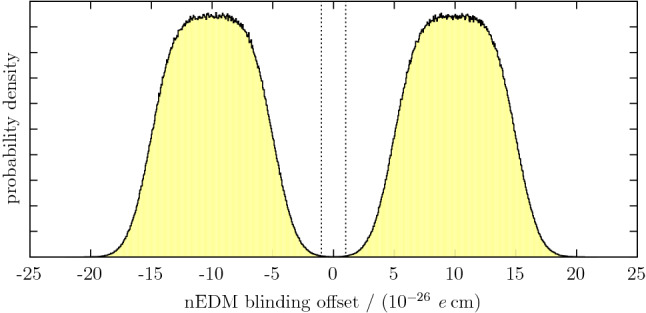
Probability density function for the choice of the blinding offset created with $10^6$ samples. The dashed vertical lines indicate the $\pm 1\sigma$ sensitivity of the data accumulated in 2015 and 2016 assuming a mean value of zero. For psychological reasons, the probability that an offset in this range is selected is kept very small but non-zero (integrated probability $\approx 2\times 10^{-4}$)

At the same time, it should be sufficiently small to guarantee that the working points are not shifted away from the steep slope of the Ramsey pattern so that the sensitivity is maintained and the Taylor expansion used in Eqs. (8)–(10) remains valid. Any error in the calculation of the number of neutrons that are shifted by the blinding process will add noise to the EDM signal, and will therefore make it more difficult to look for effects and correlations that might indicate possible systematic effects such as the motional-field effect described above.

For the nEDM experiment, four Heaviside step functions were combined to define a range of ${\pm 15\times 10^{-26}}$ *e* cm while excluding a modulus smaller than ${5\times 10^{-26}}$ *e* cm. This function was then blurred with a Gaussian of width ${\pm 1.5\times 10^{-26}}$ *e* cm to ensure that even a large analysis result could not lead to a bias, while at the same time retaining the high likelihood of having a reasonably small offset.

The extremely unlikely possibility that a value from the tail of the Gaussian that extends beyond ${\pm 1\times 10^{-24}}$ *e* cm might have been chosen was also explicitly excluded in order to ensure that the working points remained within the linear region of the original Ramsey fringe. One could argue that this latter step represents a small psychological bias, but – notwithstanding the previously existing world limit – a one-day measurement without blinding leads to the certain conclusion that the true nEDM value must be smaller. Finally, a modulus of ${< 1\times 10^{-28}}$ *e* cm was also excluded for technical reasons, since when communicating between different programs a value of exactly zero was used for cycles that should not be blinded at all, *e.g. *those with no applied electric field. Figure 3 shows the probability distribution of the blinding offset.

### Secondary blinding and reblinding

**Fig. 4:**
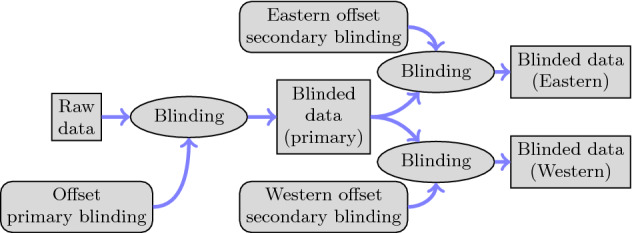
Illustration of primary and secondary blinding. Each analysis group has access only to their respective blinded data set, “Eastern” or “Western”

The nEDM collaboration decided before data taking began to have the analysis carried out by two independent teams, referred to as Eastern and Western (loosely reflecting the geographic distributions of the involved institutions). In order to allow them to communicate without introducing a bias in case of any discrepancy over the mean value of the nEDM, it was decided that in addition to the first-stage “primary” blinding the same algorithm would be used to apply a separate “secondary” blinding that was distinct for each group, *i.e. *with a different additional offset. Figure 4 illustrates this process.

During the early days of data taking some concern was expressed that the automatic fitting algorithm might not work properly in all cases, or that some important properties of the data might be hidden as a result of the blinding, or that some other similarly unexpected events might make it necessary to significantly change the blinding algorithm. In order to provide a consistent data set in any of those cases, it would be necessary to run a modified blinding program again from scratch on the raw data. However, since the first set of blinded data would by then already be available to the analysis teams, it would be trivial for them to compare two versions of the same data file, and by leaving out all mismatching events they would have an unblinded data set with a statistical significance close to the original data set. In order to avoid such a scenario it was ensured that the pseudo-random number generator delivered reproducible numbers, and that the neutrons to be transferred were selected reproducibly. Thus, if *e.g. *one version of the blinding algorithm were to shift seven neutrons and the other eight within a given cycle, the two resulting files would only differ by one neutron for that cycle. Therefore, a reblinding using the same or similar offset and a slightly modified algorithm could be carried out without danger of inadvertent unblinding. It should be noted that reblinding with an offset of opposite sign would immediately reveal both offsets.

In addition to transferring the neutrons between spin states, the blinding algorithm also marks each blinded data file with the date of blinding and the version number of the blinding code in order to ensure that those otherwise very similar files remain clearly distinguishable.

Ultimately, this reblinding feature was not used since no large discrepancies occurred within the data analysis.

### Pseudo-random number generator (PRNG)

In principle, the random numbers used should meet the same strict requirements as those for strong cryptography regarding the prediction of numbers, the correlation between them and the uniformity of their distribution. However, the quantity of random numbers required was very small – typically a dozen per cycle for about 50,000 cycles, where each cycle gets a new seed. Therefore, a prediction attack to reveal the blinding offset would be extremely unlikely to succeed even if the random number generator were not to be of the highest quality. In contrast, the quality of the generator is important in terms of non-correlation and uniformity in order to avoid the danger of introducing noise or a systematic bias to the blinded data. The standard PRNG of many computer languages, the linear congruential generator, may therefore not be suitable. Furthermore, for the reblinding it is absolutely essential that the same algorithm should remain available for a significant number of years. Thus, any libraries that might be anticipated to vary either over time or between different computers were avoided, and the decision was made to use WELL1024a [26]. The Box-Muller transform [27] was used to convert uniformly distributed to normally distributed random numbers where necessary.

The random seed for each cycle must be reproducible over a time period of years, and it needs to remain secret after blinding. The data format used, which, as noted above, is an event list of particle detection per channel, includes a periodic counter of accumulated events in every channel. This led to the choice of using a 1024 bit checksum over the last 130kByte of the unblinded file. If the data files were not to include such a counter, the blinded data file would be very similar to the unblinded one and the seed would not be secret. In such cases an alternative approach would be to use the noise in the detector for the seed creation, *e.g. *from gamma events. For the secondary blinding, the original unblinded data were used for the seed calculation. This would help if a reblinding at both primary and secondary level were ever to become necessary.

### Online blinding

In order to calculate the phase of the actual working point $\phi _i$ the blinding process requires knowledge of $\varPhi$ and $\alpha$. This information is available only after a full run has been recorded, since it arises from the overall Ramsey fit. Consequently, no blinded data are available before the end of each run. However, it is absolutely necessary to have some live data available for quality checks of the ongoing measurement. An intuitive thought would be to publish a rounded version of the neutron counts in order to disguise the blinding offset. In fact, in order to make this disguise effective the rounding must be so coarse that the number obtained would be useless as a quality check. As a solution to this problem, an online blinding mechanism was devised. For this purpose, an additional blinding offset was created randomly for each run. The range for these random numbers, which were drawn from a uniform probability distribution, was ${\pm 1\times 10^{-23}}$ *e* cm and thus was about a hundred times larger than the range of the regular blinding offset. The list of online blinding offsets used was stored in a location with restricted access, and has not been used for any other purpose than debugging the program. The online blinding algorithm does not provide a $\varPhi$ parameter; this means that it assumes a zero magnetic-field gradient for the calculation of the number of neutrons to be shifted. Furthermore, it assumed perfectly symmetric detector efficiencies and uses $\bar{N}$ instead of $N_i$. In all other aspects the algorithm was identical to that used for the regular blinding. The frequency shift introduced through the online blinding was of the same order of magnitude as those arising from changes in the magnetic field gradients, either intentionally introduced by the trim coils or by external fluctuations. With the online blinding system in place it was possible to make neutron counts available to the user immediately after each cycle, with adequate data quality to allow standard online checks to be carried out. However, these data obviously could not be used meaningfully for any further analysis.

It was important that cycles without electric field were not blinded at all, since they were used by the DAQ to choose the working points so as to have symmetric neutron count rates even in the presence of magnetic field gradients.

### Technical details

The supervisory control and data acquisition systems of the experiment were partly modular, with all processes and file handling concerning neutron counting being hosted on a dedicated computer running Linux. Time was synchronised between all computers, and control communication was done via Ethernet (TCP/IP). Thus, with simple user permissions provided by the file system it was possible to restrict access to the binary code of the blinding program, which contained the blinding offset, as well as to the raw data files. It was particularly beneficial that the computer could be started with a common unprivileged account. The DAQ program and thus the blinding process were given different permissions via the setuid bit. Consequently, the blinding process had access to the secret blinding offset and could write data files that standard users could not read.

A typical run of several days generated about a dozen gigabytes of data. With files of this size, the blinding process took several minutes. It was obviously desirable to have immediate feedback about the blinding and any potential problems, but in principle this would have meant blocking the DAQ system for significant periods of time. The blinding process was therefore split into two parts. The first part selected the data and carried out the fit of $\alpha$, which was reported to the main DAQ and thus to the user. This could be completed within a second. The process would then fork itself, on the one hand quitting to make the system available for the following run, while on the other hand simultaneously carrying out the intensive work of transferring neutron data between the two detector arms.

During data taking the blinding program was supposed to run autonomously and without intervention. This meant that it had to handle some irregular conditions:

- Data that did not contain EDM information must not be blinded; they were instead revealed immediately. These were typically runs without applied high voltage, or runs with cycles that did not have two spin-flips. Such measurements occurred fairly frequently in order to characterise the UCN source [28, 29], the detector, or the background.
- The fitting process ignored single cycles with a low neutron count rate. A lower threshold of 1000 counts was chosen, since such a low count rate would not be used for nEDM analysis.
- Cycles with an unphysically high count rate were not blinded, since these could effectively disclose the blinding offset. An upper threshold of 50,000 neutrons per cycle was chosen; this was a factor of ten greater than the actual numbers observed during commissioning, and nearly a factor of three greater than the genuine maximum observed.
- Blinding was only automatically applied if the quality of the Ramsey fit was sufficiently good. The threshold here was $\chi _\text {red.}^2 < 3$.

In case of doubt the blinding process neither blinded nor revealed data, but rather made a request via E-mail for human intervention.

#### Manual interventions

Great care was taken during the design of the blinding algorithm to minimize the need for human intervention during data taking. This required automatic handling of unusual circumstances with respect to data quality or malfunctions of parts of the apparatus. Inevitably, due to the complexity of the experiment, some manual interventions during data taking were necessary. In these circumstances the data were assessed by the blinding coordinator in order to either reject bad cycles and to apply the blinding on the remaining cycles of the run, or to divide a run into pieces between magnetic field jumps and to apply the blinding process separately on these parts. Between 13 September 2015 and 21 December 2016 inclusive, 1072 runs with neutron data were recorded. Of these, 113 runs were automatically blinded and 14 runs needed manual blinding. All of these contained information on the EDM. The remaining 945 runs were usually much shorter and were used for calibration, setup, and systemic studies of the apparatus, often undertaken when no neutrons were available. Of this set, 925 runs were revealed promptly, while 20 runs needed manual revealing. Notably, not a single run () was blinded or revealed automatically where not intended, while more than 96% were treated automatically, and therefore were not subject to any delay.

#### Secrecy

While no malign intent is assumed, there are a number of scenarios under which the blinding offset – a single simple number – could inadvertently be revealed if the raw data were not adequately protected:

- During the data analysis process there may be a temptation to carry out a test that would be simpler to run on the unblinded raw data.
- Obtaining access to “forbidden” data will always of itself be tempting to some, merely as a challenge or puzzle that they wish to demonstrate they can solve.
- Others may seek the “codebreaking” challenge of attempting to decrypt data or of applying statistical attacks on them.

With this in mind, the blinding offset was stored using asymmetric encryption with the public part of an RSA-key pair directly after it had been created. The blinding offset together with some metadata only amounts to 192 bits; thus, a simple asymmetric encryption is possible. The private key to decrypt the blinding offset was injected into the executable of the blinding program at compile time. Access to the executable program is restricted by file system permissions. The original private key was stored with password RSA encryption using OpenSSL and was thus only available to the blinding coordinator. Access to data files was restricted by file system permissions.

These cryptographic and organisational measures were deemed reasonable in order to prevent accidental unblinding of the data. They were easy to implement and did not have any impact on permitted workflows. Although fairly robust, they are certainly not sufficient to protect against either physical theft of hard drives or manipulation of software with malicious intent. Any further protection would require the restriction of physical access to the DAQ computer or its boot process. Encryption of the operation system via a Trusted Platform Module chip is nowadays available and would suffice for this task. However, this could potentially have had an impact on the maintainability of the system, especially in case of hardware problems. The existing hurdles were therefore considered to be sufficiently high.

### Effects of noise and asymmetry

The blinding algorithm manipulates the data, including with the use of random numbers and fit results. This procedure naturally introduces some noise. In this section we discuss the level of this noise and the resulting consequences.

#### Noise from fractional neutron numbers

In Sect. 3.2.1 we described how a random number (normal distribution with $\sigma =2$) is added to the fractional number of neutrons to be transferred before rounding to an integer value. Solving Eq. (11) for *d* allows one to calculate how much noise is added to the final nEDM result due to this additional random process. Using the average number of neutrons per cycle $\overline{N} = 11{,}400$, the average visibility $\overline{\left| \alpha \right| } = 0.75$, and the applied electric field $E={11}\,{\hbox {kV/cm}}$, the additional noise amounts to ${7.7\times 10^{-26}}$ *e* cm per cycle. The additional statistical uncertainty for the mean of all 54068 cycles is ${3.3\times 10^{-28}}$ *e* cm, which is about 3% of the uncertainty due to counting statistics.

#### Noise from detector asymmetry

In Sect. 3.2.2 we described the determination of $\alpha$ and $A_m$ through fitting. These quantities each have their own statistical uncertainty. The mean of the fit value of the visibility $\left| \alpha \right|$ was 0.75, and the mean of its uncertainty was 0.003. The mean values of the detector asymmetry were $A_{\mathrm {N}}=0.032$ and $A_{\mathrm {I}}=-0.036$ in 2015, both with a standard deviation of 0.002. In 2016 the mean was $\left| A_m\right| =0.004$ with a standard deviation of 0.001. The mean of the individual uncertainties within each run was always below 0.001. Thus in 2015 there was a significant asymmetry. The number of neutrons to be transferred is calculated from these numbers via Eqs. (17) and (11). At our working points the result of $\sin (\arccos (x))$ lies between 0.98 and 0.99 for any *x*. Thus no matter how large the fluctuations of $A_m$ may have been, the resulting noise on $\delta N$ is less than 1% and is therefore also negligible, being significantly smaller than the noise arising from the integer rounding described in Sect. 3.8.1.

#### Noise from visibility

The parameter $\alpha$ enters directly in Eq. (11), but as the observed relative uncertainty $\frac{0.003}{0.74}=0.004$ is also very small, the contribution to the noise is once again negligible.

#### Noise from neutron number per cycle

The final parameter in Eq. (11) is the measured quantity $N_i$. Despite being a noisy observable, it does not contribute to any noise in the blinding since it represents the exact value of the number of neutrons for this particular cycle.

#### Verification on test data

The very earliest data, a set of 24 runs obtained prior to 13 September 2015, were taken with an early implementation of the blinding process. In order to test the blinding process with real data, this subset of the data was made available to the analysis teams both with and without a blinding offset of $d^*={+1.951\times 10^{-25}}$ *e* cm. (Note that the blinded sample was not used in the analysis presented in Ref. [5].) These runs each have an irreducible statistical sensitivity that ranges from ${0.9\times 10^{-25}}$ *e* cm to ${2.4\times 10^{-25}}$ *e* cm, accumulating to a total of ${3.2\times 10^{-26}}$ *e* cm. The data were analysed twice. The first time – with what was then still a fairly rudimentary data analysis – was in September 2015, just prior to the decision to continue with the full implementation of the blinding. The second occurrence used an almost final analysis. Both tests showed that the blinding algorithm increased the uncertainty by ${2\times 10^{-28}}$ *e* cm, corresponding to 0.5% of the statistical sensitivity of the data set. The blinding offset predicted by the analysis matched the applied offset to within ${0.2\times 10^{-26}}$ *e* cm, which was a tenth of the uncertainty of the analysis. This comparison was carried out before removal of the secondary blinding of the full data set in order to provide a metric to assist in judging the relative quality of the analyses. After unblinding, this test was repeated with the full data set as described in the next section.

## Unblinding

Each data analysis team worked on a doubly (primary + secondary) blinded data set, and ultimately extracted their own estimator for the blinded nEDM value and its uncertainty. Once the collaboration was convinced that these analyses were complete, a comparison based on appropriate parameters and distributions was undertaken. One comparator was, for example, the nEDM uncertainty. Moreover, after grouping the data in sequences (sets of cycles for which the magnetic field, in particular, did not change – and thus, normally one or several successive runs) it was possible to check, sequence by sequence, the difference between the extracted nEDM and its mean value (averaged over all sequences). This difference was useful to check that the two analysis results showed the same correlations with respect to external parameters. In particular, the measured neutron EDM is shifted from the true value of the neutron EDM by a systematic effect that is linear in the vertical gradient, and the analyses are designed to correct for this correlation [30].

The decision to proceed to the first unblinding step, which consisted of removing the secondary blinding offsets, was taken based on the agreement of all comparators.

After this first unblinding it was possible to cross-check the two analyses with respect to the secondary blinding offset, the results of which are shown in Table 1.

**Table 1:** Estimators of the neutron EDM and their statistical uncertainties derived by the two analysis teams, in units of ${\times 10^{-26}}$ *e* cm

	Western		Eastern	
nEDM estimator	Value	$\chi ^2/N_{\text {dof}}$	Value	$\chi ^2/N_{\text {dof}}$
Doubly blinded $\tilde{\tilde{d}}$	${15.39}\,{\pm 1.07}$	90.7/86	${3.80}\,{\pm 1.11}$	91.2/86
Singly blinded $\tilde{d}$	${5.97}\,{\pm 1.07}$	93.0/86	${6.15}\,{\pm 1.11}$	93.2/86
Non-blinded *d*	${-0.02}\,{\pm 1.07}$	92.5/86	${0.16}\,{\pm 1.11}$	92.4/86
$\tilde{\tilde{d}} - \tilde{d}$	9.43		$-2.35$	
Input offset $d^{\prime \prime }$	9.48		$-2.33$	
Difference $\tilde{\tilde{d}} - \tilde{d} -d^{\prime \prime }$	$-0.05$		$-0.02$	
$\tilde{d} - d$	5.99		5.99	
Input offset $d^{\prime }$	6.02		6.02	
Difference $\tilde{d} - d -d^{\prime }$	$-0.03$		$-0.03$	

is the estimator of the doubly blinded data, while  is the estimator of the singly blinded data. The input offset $d^{\prime \prime }$ is the value of the secondary blinding offset, which was de-encrypted during the first, relative, unblinding on 23 October 2019. The input offset $d^{\prime }$ is the value of the primary blinding offset, which was de-encrypted during the second unblinding on 28 November 2019. All analysis results in this table arise only from data taken after 13 September 2015; data prior to this were not blinded with the same offsets and thus cannot be compared. Consequently, the value *d* listed here differs slightly from the final result [5]. The observed span of $\chi ^2$ values of 1.8 corresponds to a change of uncertainty of $1\times 10^{-28}$ *e* cm. The fluctuation in this range – even to smaller values – is within statistical expectation

This allowed a direct comparison of the (singly blinded) nEDM values obtained by the two teams. If any discrepancy had been found, a longer and detailed comparison would have had to have been carried out at this point. Should this have become necessary, possible approaches that were discussed included (a) running both analysis codes on a common subset of data and converging parameters and code, *i.e. *cut criteria and methods, until the results matched, and (b) producing new sets of secondary blinded data, although this would have been of limited use since by then both analysis teams would implicitly know their offsets, and (c) producing an alternative blinded data set directly from the original raw data with a new unknown random offset.

**Fig. 5:**
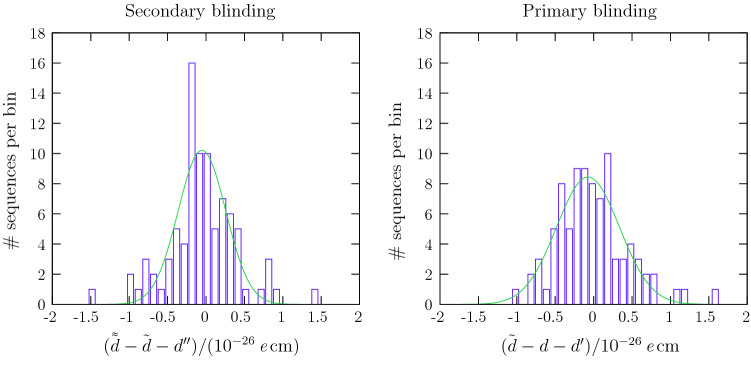
Difference between results of the analysed blinded and unblinded data sets and the corresponding offsets, shown separately for each of the two blinding steps. The bin width is $10^{-27}$ *e* cm. Both peaks are centred well within $10^{-27}$ *e* cm. Only results from the Western analysis using data taken after 13 September 2015 are shown; the Eastern analysis yields similar results

Since the two analysis teams were in agreement, it was possible (once it had finally been confirmed that all known systematic effects had been evaluated) to proceed directly to the removal of the primary blinding. The offset was therefore revealed and subtracted, to yield a true nEDM estimator. In addition, the same analysis codes together with the same settings, *e.g. *for cuts, were applied to the original, non-blinded data set, which had been kept hidden up until that point. The result of the direct analysis of this non-blinded data set was of course expected to match with that emerging from the analysis of the blinded set minus the applied blinding offset. From theoretical estimation, as well as from the experience with the early data taken without blinding, agreement between these two approaches had been expected to be at the level of $10^{-27}$ *e* cm. In the posterior comparison, as shown in Table 1, this was confirmed perfectly. Fig. 5 shows a comparison between the injected blinding offset and the one predicted by the analysis from the Western team. The non-zero width of the peaks indicates that the blinding algorithm does indeed, as expected from Sect. 3.8.1, inject some noise into the individual sequences or cycles. The widths of the Gaussians fitted to the distribution were $0.31(5)\times 10^{-26}$ *e* cm and $0.41(4)\times 10^{-26}$ *e* cm for secondary and primary blinding, respectively. The sequences consisted of 514 cycles on average. Thus, the observed widths are compatible with the expected uncertainty of the mean due to the noise of $0.34\times 10^{-26}$ *e* cm.

The agreement of the difference ‘analysis of blinded data’ minus ‘analysis of non-blinded data’ with the blinded offset is outstanding.

## Costs and benefits

As discussed in Ref. [31], blinding does not in general come without cost. For the method presented here, the costs were primarily the manpower required for design, implementation and study of the technique. A small amount of statistical noise was introduced into the blinded data, but this tiny contribution was only present in the blinded data sets; it left the final analysis unaffected. Ultimately, the method described here did not suffer from the various costs that have typically been present for other blinding techniques – for example, all analysis channels were immediately available and no signals or features, other than the true nEDM itself, were hidden. It is notable that the blinding even permitted the analysis of a periodically changing nEDM [32] without revealing the unblinded result of the static signal.

Most importantly, the blinding provided a very substantial benefit to the nEDM analysis, and not only in that it eliminated the effects of an unconscious bias. Since in past measurements the true nEDM results have always been indistinguishable from zero they were sign insensitive, and as such also insensitive to potential sign errors in the analysis. However, in this case the false signal in the blinded data had a value significantly away from zero, and thus included a clearly identifiable sign. This sign showed up in the various analysis channels, *e.g. *with its dependence upon magnetic-field gradients, and as such at one point it actually revealed a mistake in an early version of our data analysis when the code was tested with a known nEDM offset.

## Possible improvements

In order to handle the non-integer number of neutrons to be transferred in each single cycle, a normally distributed random number of width 2 was used as described in Sect. 3.2.1. Future implementations will use a rectangular probability density function of width 1. This will provide perfect linearity and will reduce the introduced noise by a factor of two, down to the intrinsic minimum.

## Summary and conclusion

For the first time, a blinding technique has been developed for and applied to a neutron EDM measurement. The true EDM value is hidden by an offset, while other variables of interest are unaffected. The algorithm presented modifies only a copy of the recorded data, and saves the original data in a hidden location. Secondary blinding and the possibility of re-blinding are innovations that further reduce risks that are often associated with blinding. The artificial increase in noise in the blinded data sets as a result of this process has been shown to be negligibly small, and disappears automatically in the final result.

## Data Availability

This manuscript has associated data in a data repository. [Authors’ comment: The experimental data will be published at a later point.]
